# The Emotional Experiences of Women Suffering From Premenstrual Dysphoric Disorder in the United Kingdom

**DOI:** 10.1192/bjo.2024.103

**Published:** 2024-08-01

**Authors:** Nasara Al-Hassan, Carinna Hockham, Lynsay Matthews, Robyn Norton, Kate Womersley

**Affiliations:** 1 Imperial College London, London, United Kingdom; 2 University of West of Scotland, Paisley, United Kingdom

## Abstract

**Aims:**

1. To hear directly from women suffering from PMDD about their lived experiences of PMDD and the impacts that it has on their daily lives.
2. To raise awareness about the impacts that PMDD can have on patients' quality of life, relationships, and productivity, to improve clinicians’ understanding of patients' needs.
3. To identify a gap in research into PMDD within the UK and highlight the need for further research.
4. To improve awareness of PMDD amongst diverse stakeholders, including women who are not yet diagnosed with PMDD, employers, and policymakers.

**Methods:**

Participants were recruited from the UK's PMDD Patient Insight Group and screened using the Premenstrual Symptom Screening Tool (PSST) for PMDD. Eligible participants were purposively sampled, and 15 participants were invited to a semi-structured scheduled interview on Zoom. Interviews were transcribed using NVivo transcription software, and inductively analyzed using reflexive thematic analysis in NVivo 14.

**Results:**

Thirteen subthemes were identified and organised around four main themes: Theme 1: ‘Jekyll and Hyde’, Life with PMDD, Theme 2: ‘The Aftermath’, The Impact of Living with PMDD, Theme 3: ‘Surviving PMDD’, Coping Strategies, and Theme 4: ‘Seeking Treatment’, Experiences with Healthcare. The themes identified in this study highlight the negative experiences of women living with debilitating symptoms that appear during the luteal phase and disappear following the onset of menstruation. Themes also capture the immense burden PMDD places on a sufferer by uncovering how exactly these symptoms affect interpersonal relationships, career progression, quality of education received, and relationship with oneself. Theme 4 focuses on women's negative experiences with healthcare stemming from a lack of awareness of PMDD in the medical community.

**Conclusion:**

The findings of this study highlight the critical importance of understanding the contextualized experiences of women living with PMDD in the UK and bringing to light the immense monthly burden sufferers face. To prevent women and Assigned Female At Birth (AFAB) individuals from experiencing severe and prolonged psychological distress which can have fatal consequences, there needs to be greater understanding and awareness of PMDD in both medical and lay communities. In addition to this, clinicians must be trained in PMDD assessment and research should be encouraged to introduce new treatments and to implement policies that minimize the burden of PMDD in the workplace.

